# Inference of Evolutionary Forces Acting on Human Biological Pathways

**DOI:** 10.1093/gbe/evv083

**Published:** 2015-05-13

**Authors:** Josephine T. Daub, Isabelle Dupanloup, Marc Robinson-Rechavi, Laurent Excoffier

**Affiliations:** ^1^CMPG, Institute of Ecology and Evolution, University of Berne, Switzerland; ^2^Swiss Institute of Bioinformatics SIB, Lausanne, Switzerland; ^3^Department of Ecology and Evolution, University of Lausanne, Switzerland; ^4^ Present address: Institute of Evolutionary Biology (UPF-CSIC), Barcelona, Spain

**Keywords:** human evolution, pathway analysis, polygenic selection, McDonald–Kreitman test

## Abstract

Because natural selection is likely to act on multiple genes underlying a given phenotypic trait, we study here the potential effect of ongoing and past selection on the genetic diversity of human biological pathways. We first show that genes included in gene sets are generally under stronger selective constraints than other genes and that their evolutionary response is correlated. We then introduce a new procedure to detect selection at the pathway level based on a decomposition of the classical McDonald–Kreitman test extended to multiple genes. This new test, called 2DNS, detects outlier gene sets and takes into account past demographic effects and evolutionary constraints specific to gene sets. Selective forces acting on gene sets can be easily identified by a mere visual inspection of the position of the gene sets relative to their two-dimensional null distribution. We thus find several outlier gene sets that show signals of positive, balancing, or purifying selection but also others showing an ancient relaxation of selective constraints. The principle of the 2DNS test can also be applied to other genomic contrasts. For instance, the comparison of patterns of polymorphisms private to African and non-African populations reveals that most pathways show a higher proportion of nonsynonymous mutations in non-Africans than in Africans, potentially due to different demographic histories and selective pressures.

## Introduction

In the search for genomic signals of natural selection (e.g., reviewed in Nielsen 2005), there has been a recent shift from single gene to gene set approaches, where the focus moved to gene networks, pathways, and interacting genes (Daub et al. 2013; Fraser 2013; Zhang et al. 2013; Berg and Coop 2014). Studying groups of functionally related genes makes biological sense for several reasons. First, as selection is acting on phenotypic traits usually controlled by many genes (Stranger et al. 2011), we would expect it to affect multiple genes or a whole pathway rather than a single gene. A biological pathway or a gene network is therefore a more natural unit for selection tests. Second, mutations in one gene can induce the modification of functionally connected genes to adapt to or compensate for the initial change (Pazos and Valencia 2008), and such co-evolution can lead to a cascade of evolutionary changes within a gene network. Third, many small effect mutations can together have a large effect on polygenic traits. It has been suggested that selection on such traits, termed polygenic selection, usually acts on standing variation at several loci at the same time. In the long run, this would lead to an increase in the frequency of a suitable combination of alleles that control the favored phenotype (Pritchard et al. 2010).

For example, using levels of population differentiation between human groups, we have recently found several pathways involved in immune response to have been under positive selection in recent human history (Daub et al. 2013). In this article, we investigate whether and what type of polygenic selection could have acted at different stages of human evolution, by comparing patterns of diversity between humans and chimpanzees. The rationale is that since selective pressures could have changed over time for some biological pathways, recent or old episodes of selection cannot be detected by only looking at current human diversity. Our method contrasts patterns of fixed and polymorphic mutations in humans, allowing us to detect various selective pressures having affected our species at different periods of its evolution.

Our approach is based on statistics previously used in the classical McDonald–Kreitman (MDK) test for positive selection (McDonald and Kreitman 1991), a test that compares the ratio of the number of nonsynonymous substitutions to synonymous substitutions between species (DN/DS) to the same ratio within species (PN/PS). Assuming that synonymous mutations are neutral, a higher DN/DS than PN/PS ratio is expected in case of ancient adaptive selection, because positively selected mutations rising to fixation in a population would contribute more to divergence than to polymorphism. The related statistic α=1-(DS×PN)/(DN×PS) has been used to estimate the proportion (α) of adaptive nonsynonymous substitutions in a genome (Smith and Eyre-Walker 2002). A limitation is that the same α value can be obtained for different combinations of PN/PS and DN/DS ratios. To account for this problem, we introduce here a testing procedure, based on a two-dimensional (2D) decomposition of α, which identifies gene sets departing from a genome-wide null distribution and leads to a better interpretation of the results by a visual inspection of bivariate distributions.

## Materials and Methods

### Data Handling and Collection

#### Ensembl Gene Data

We downloaded the exon coordinates of protein coding human genes from Ensembl (Ensembl version 64, September 2011, Flicek et al. 2014). We then only considered those genes (and their corresponding coding exons) that have a chimp ortholog with a “known” status as defined in Ensembl version 64, and genes having a chimp ortholog without known status but with a known mouse ortholog. The second group represents genes that most probably have a real ortholog in chimp but are not annotated as such (yet) due to the lower quality of the chimp sequence. This left us with 18,078 genes and we will refer to this set as *G*_Ensembl_. We computed the total exon length of each gene by summing the length of all its coding exons but only counted a site once if it was part of two overlapping exons. We thus used the longest coding exon definition for each gene.

#### Human Single-Nucleotide Polymorphisms

**Fig. 1.—:**
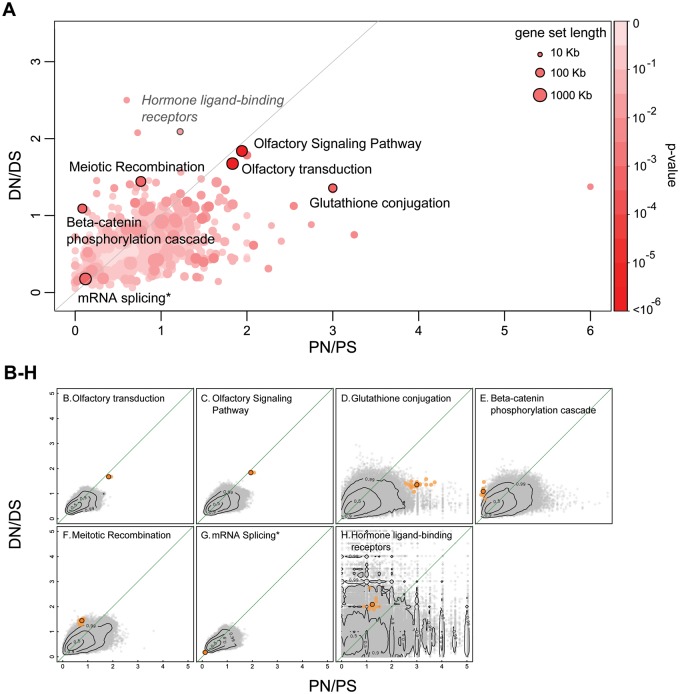
Results from the 2DNS test. Here, shared SNPs (SNPs that are polymorphic both in African and non-African populations) are compared with fixed substitutions in the human branch. (*A*) Distribution of DN/DS and PN/PS ratios of all tested pathways. Each dot represents a pathway with color corresponding to its significance and size to its total exon length. The six pathways that scored significant (*q* value < 0.2; table 2) in the 2DNS test are highlighted by a black circle. Note that seemingly outlier gene sets may not reach significance due to their small size, which increases the variance of their null distribution. For example, the *Hormone ligand-binding receptors* pathway (depicted in gray; *H*), has a high DN/DS ratio, but because of its small size (ten genes, 24 kb total exon length) its null distribution is very widespread in the 2D space. (*B–G*) Null distributions for six significant (*q* < 0.2) pathways. (*H*) Null distribution for the *Hormone ligand-binding receptors* pathway as a typical example of a nonsignificant outlier. The observed positions of gene sets are indicated as orange dots highlighted by a black circle in the DN/DS–PN/PS plane, whereas the empirical null distribution is shown as gray dots (*N* = 400,000). Light-orange dots show the scores of the jackknifed gene sets. The contour lines mark the proportion (0.5, 0.9, and 0.99) of the null distribution that falls within these areas.

**Fig. 2.—:**
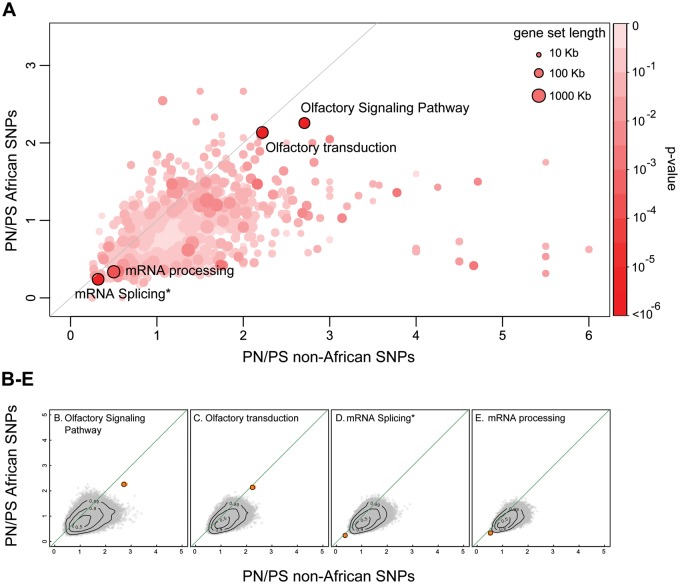
Results of the 2DNS test on PN/PS ratios of all tested pathways. Here, non-African SNPs (SNPs that are only polymorphic in non-African populations) are compared with African SNPs (SNPs unique to African populations). (*A*) Distribution of DN/DS and PN/PS ratios of all tested pathways. Each dot represents a pathway with color corresponding to its significance and size to its total exon length. The four pathways that scored significant (*q* value < 0.2) in the 2DNS test are highlighted by a black circle. (*B–E*) Null distributions for the four significant (*q* < 0.2) pathways. The observed positions of gene sets are indicated as orange dots highlighted by a black circle in the DN/DS-PN/PS plane, whereas the empirical null distribution (*N* = 400,000) is shown as gray dots. Light-orange dots show the scores of the jackknifed gene sets. The contour lines mark the proportion (0.5, 0.9, and 0.99) of the null distribution that falls within these areas.

Human single-nucleotide polymorphisms (SNPs) were inferred from the comparison of the whole genomes of 42 unrelated individuals sequenced by Complete Genomics (CG) at a depth of 51–89X coverage per genome (Drmanac et al. 2010). The 42 individuals were sampled in 3 African populations (four Luhya from Webuye, Kenya; four Maasai from Kinyawa, Kenya; and nine Yoruba from Ibadan, Nigeria), and 5 non-African populations (nine Utah residents with Northern and Western European ancestry from the CEPH collection; four Han Chinese from Beijing; four Gujarati Indians from Houston, TX; four Japanese from Tokyo; and four Toscans from Italy). The SNPs were divided into three categories: Sites which are polymorphic in Africans only (African SNPs), sites polymorphic in non-Africans only (non-African SNPs), and sites polymorphic in both Africans and non-Africans (shared SNPs). The shared SNPs presumably arose before the migration of modern humans out of Africa and are therefore depleted from recent deleterious mutations that otherwise could distort selective signals. This group of SNPs was used in the 2DNS test (fig. 1 and supplementary fig. S1, Supplementary Material online). The African and non-African SNPs were used in a further analysis to compare demographic patterns between the two regions (fig. 2).

To investigate the effect of larger sample sizes on our results, polymorphism data were also obtained from the 1000 Genomes project phase 3 (Abecasis et al. 2012, hereafter called the 1000G set), including 288,003 SNPs from 1,594 individuals sampled from 16 nonadmixed populations (five from Africa, seven from Asia, and four from Europe, see supplementary table S1, Supplementary Material online). Note that the 1000G SNP set consists of polymorphisms called from a combination of both low and high coverage data (between 8X and 30X) and that it only targets about 40% of the exome, whereas CG covers the whole exome at higher coverage (51–89X). For these reasons, the CG set was used in the main analyses. In the analyses where the CG and 1000G datasets where compared, we only considered SNPs that were in exonic regions both targeted by the 1000G project and fully sequenced in all 42 CG samples.

#### Human Chimp Substitutions

Substitutions between human and chimp were inferred from the comparison between the reference genomes of the two species, using the syntenic net alignments between hg19 and panTro2 available on the University of California–Santa Cruz (UCSC) platform (Karolchik et al. 2012). The inferred mutations were then placed on the human-specific or chimp-specific branches of the tree by comparing the nucleotides observed in human and chimp to the orthologous base in the ancestral sequence derived from Ensembl Compara release 59 and used by the 1000 Genomes consortium (Abecasis et al. 2012); only mutations which could be placed unambiguously on the human or chimp branch were used.

#### Mapping and Annotation of Mutations

We mapped the mutations to the coding exons of the human genes in the *G*_Ensembl_ set. Our polymorphism and divergence data cover a largely overlapping part of the exome: 89.2% of the exome is fully sequenced in all 42 CG samples, whereas the human–chimp alignment covers 96.3% of the exome, resulting in 86.8% of the exome covered by both datasets.

We classified the mutations as either synonymous or nonsynonymous using ANNOVAR (Wang et al. 2010). As a measure of evolutionary constraint, the sites were assigned GERP scores (Cooper et al. 2005) collected from the UCSC platform (Karolchik et al. 2012), which are estimated with the GERP++ method (Davydov et al. 2010).

#### Ensembl to Entrez Gene ID Conversion

As genes in Biosystems gene sets are annotated with Entrez gene IDs, we mapped the Ensembl gene IDs in *G*_Ensembl_ to Entrez IDs by constructing a one-to-one Ensembl–Entrez conversion table. To do this, we first downloaded from the National Center for Biotechnology Information (NCBI) Entrez Gene website (Maglott et al. 2011) a gene list (*G*_Entrez_) with 19,759 “current” protein coding human genes (http://www.ncbi.nlm.nih.gov/gene, accessed on February 7, 2013). Next, we collected conversion tables (containing one-to-many or many-many mappings) from three sources: Ensembl (version 64, September 2011), NCBI (ftp://ftp.ncbi.nih.gov/gene/DATA, accessed on February 7, 2013), and HGNC (http://www.genenames.org/biomart/, accessed on September 3, 2012). From these tables, we only kept rows with genes in *G*_Ensembl_ and *G*_Entrez_. We then pooled the three tables and counted the occurrences of each unique Ensembl ID–Entrez ID combination. For each Ensembl ID, we kept the Ensembl–Entrez match with the highest count (in case of multiple options, we took the first one) and repeated this for each Entrez ID. The resulting table contained 17,474 one-to-one Ensembl to Entrez gene ID conversions and we used the genes from this table (gene list *G*) for our further analyses.

#### Gene Sets

We downloaded 2,402 human gene sets from the NCBI Biosystems database (Geer et al. 2010) (http://www.ncbi.nlm.nih.gov/biosystems, accessed on February 2, 2013). The Biosystems database is a repository of gene sets collected from other pathway databases, such as BioCyc (Caspi et al. 2014), KEGG (Kanehisa and Goto 2000; Kanehisa et al. 2014), The National Cancer Institute Pathway Interaction Database (Schaefer et al. 2009), Reactome (Croft et al. 2014), and Wikipathways (Kelder et al. 2012). A gene set represents a group of genes involved in biochemical interactions that underlie a biological process. Examples of gene sets include metabolic pathways, gene regulatory networks, or signal transduction pathways.

We excluded genes that could not be mapped to the gene list *G* and removed gene sets with less than ten genes. Furthermore, we identified 95 groups of (nearly) identical gene sets, namely sets sharing at least 95% of their genes, and replaced each group by a union gene set (in the text their name is followed by “*”) consisting of all genes in that group. The remaining collection of 1,366 gene sets (*S*) was used for our analyses. Note that, for ease of reading, we use both the terms pathway and gene set interchangeably throughout the main text; strictly only a small proportion (<2%) of the sets in *S* are labeled as “complex” rather than “pathway” in the Biosystems database.

### Data Analysis

#### Test of Evolutionary Similarities between Genes of a Given Gene Set

To assess whether genes in sets tend to share the same evolutionary properties, we tested for significant differences in gene-level DN/DS (or PN/PS) ratios between gene sets. First, we estimated the variance component due to differences among gene sets (${\text{σ}}_{\text{A}}^{2}$) for all 1,366 sets using an analysis of variance (ANOVA) framework (See supplementary text S1, Supplementary Material online). We constructed the expected null distribution of ${\text{σ}}_{\text{A}}^{2}$ under the hypothesis that there are no differences between groups, by repeatedly (*n* = 10,000) recalculating ${\text{σ}}_{\text{A}}^{2}$ after permuting DN/DS and PN/PS ratios among genes. Next, to assess that the significance was not due to one or a few outlier gene sets, we repeatedly (*N* = 10,000) sampled randomly 100 sets from the list of 1,366 gene sets and computed ${\text{σ}}_{\text{A}}^{2}$ on these 100 gene sets only. We built the expected null distribution by performing the same sample procedure but permuting DN/DS and PN/PS ratios among genes for each drawing of 100 genes sets. (See also supplementary fig. S2, Supplementary Material online.) Note that the fact that genes can occur in multiple gene sets has no impact on the results, as we keep the gene set definitions fixed when creating the null distribution. Therefore, the amount of overlap between gene sets is the same in both null and observed distributions.

Note that we excluded 4,446 (2,963) genes that had zero DS (PS), leading to undefined *N*/*S* ratios, when comparing DN/DS (or PN/PS) ratios between genes (as shown in supplementary fig. S3, Supplementary Material online, and in the ANOVA test described above). In the other analyses, we could use all 17,474 genes of our gene list *G* to compute DN/DS (or PN/PS) ratios at the gene set level.

#### Gene and Gene Set Level DN/DS and PN/PS Ratios

To calculate the DN/DS ratio for a gene set, we summed up separately the nonsynonymous and synonymous fixed mutations found in all genes belonging to a given gene set (supplementary fig. S1, Supplementary Material online) and took their ratio as

(1) $${\left(\frac{\text{DN}}{\text{DS}}\right)}_{\text{set}}=\frac{{\text{DN}}_{\text{set}}}{{\text{DS}}_{\text{set}}}=\frac{\sum_{g\in \text{set}}{\text{DN}}_{g}}{\sum_{g\in \text{set}}{\text{DS}}_{g}}$$

The PN/PS ratio was calculated in a similar way. In a few cases where the same mutation occurred in more than one gene of the same gene set (due to gene overlap or exon sharing, around 0.1% of the mutations), it was counted only once.

### Null Distributions

#### Gene Set Properties Taken into Account When Building Empirical Null Distributions

We constructed empirical null distributions of DN/DS and PN/PS ratios (in short “*N*/*S* ratios”) by creating a series of random gene sets and recomputing new statistics on these gene sets. As genes in gene sets might have specific evolutionary properties, randomly sampling genes from the gene list *G* would not create a representative null distribution. Instead, we took into account the negative correlation found between the number of gene sets a given gene belongs to and the gene *N*/*S* ratio. We also took into account the fact that genes belonging to the same pathway tend to share the same evolutionary regime (supplementary fig. S2, Supplementary Material online). To preserve the evolutionary properties of gene sets, we applied a bootstrap sampling method, where we sampled genes from unions of gene sets that share a given proportion of their genes (see next section).

In addition, we also considered the fact that gene sets with a large “set length” (defined as the total exon length of its genes) accumulate on average more mutations and for this reason show a lower variance in *N*/*S* rates. We therefore created null distributions for different set length categories and tested each pathway against the appropriate null distribution.

#### Construction of Empirical Bootstrap Null Distributions

In short, we first created random gene sets of different sizes (number of genes), where we sampled the size according to the real size distribution and next computed the set length of the random sets and assigned them to different set length bins.

We created a random gene set as follows (supplementary fig. S4, Supplementary Material online). First, we sampled a gene set *S*′ from the collection *S* of all gene sets. For each sampled gene set *S*′, we collected all genes that are the union of genes in *S*′ and genes in any set *S*″ that overlaps with *S*′, in the sense that their Jaccard similarity coefficient (defined as [*S*′ ∩ *S*″]/[*S*′ ⋃ *S*″]) is larger than 0.25. Next, we drew a gene set size *k* from the geometric distribution that approximated the real gene set size distribution. If the union contained less than min(250, *k*) genes, it was discarded, otherwise we randomly sampled *k* genes (without replacement if *k* < 250, otherwise with replacement) from this union to form a random gene set. We repeated this procedure 75 × *n* (*n* = 400,000) times. For each observed and random gene set, we calculated its set length and distributed the sets according to their set length over 75 equally sized bins, explaining the factor 75 in the previous sentence. Note that we drew *k* from a geometric distribution that approximated the real size distribution but allowed for sampling gene sets smaller than ten (the minimum gene set size) to obtain sufficient sets to fill the smallest set length bins

(2) X∼Geom(p=1/(mean(set size)−10))+5

In very few occasions, the *N*/*S* ratio of a (random) gene set was not defined, because *S* was zero. In those cases, we removed the gene set from the null distribution or from the group of sets that were tested. To still have bins of size *n*, we therefore actually created 75 × *n* × 1.1 gene sets, removed the sets that did not have a defined *N*/*S* ratio, and took the first *n* remaining gene sets for each bin.

Changing the values for parameters such as the similarity coefficient or the number of bins gives similar test results. See supplementary text S2 and table S2, Supplementary Material online, for a detailed description of the parameters used and the effect of changing their values.

#### Alternative Null Distributions without Gene Set Properties

To investigate the effect of not taking into account some or all the properties of gene sets when creating a null distribution, we used several alternative sampling schemes consisting of 1) randomly sampling from the whole gene list *G*; 2) sampling from all genes that are part of at least one gene set; and 3) sampling genes with a probability proportional to the number of gene set a gene belongs to. Similar to the empirical bootstrap null distribution described above, we corrected in all cases for gene set length, by creating null distributions for specific set lengths and testing a pathway against the corresponding null distribution. Results based on these alternative null distributions are shown in supplementary figure S5, Supplementary Material online.

### Tests of Selection

#### A New Test of Polygenic Selection (2DNS Test)

We developed a new test for polygenic selection (named 2DNS), which can be regarded as a decomposition of the constitutive elements of a MDK test, DN/DS, and PN/PS. Our 2DNS test aims at detecting gene sets that are outliers in the two dimensional DN/DS − PN/PS plane relative to an empirical null distribution of joint DN/DS and PN/PS ratios. The *P* value of a gene set is estimated from the joint *N*/*S* density on a two dimensional grid. We first used the R function *kde2d* (taking bandwidth = 0.15, maximum grid size = 10 × 10 and grid point distance = 0.05) to estimate the probability density of each of the grid points. We then bilinearly interpolated the *N*/*S* coordinates of the observed gene set on the grid of the null distribution and the corresponding density with the R function *interp.surface*. The *P* value of the gene set is finally calculated by integrating over the densities of grid points that have the same or lower density as the density of the observed gene set (supplementary fig. S6, Supplementary Material online). Note that because we use a kernel density estimation, *P* values much smaller than the inverse of number of elements in the null distribution can be obtained. See also supplementary text S2 and table S2, Supplementary Material online, for a description of parameters used in the *kde2d* function and how they affect the results when their value is changed.

#### Correction for Multiple Tests

We corrected the *P* values for multiple testing by calculating the *q* value of each gene set using the function *qvalue* from the R package *qvalue* with default settings. The *q* value of a test measures the minimum proportion of false positives (the false discovery rate, FDR) when that test is called significant (Storey and Tibshirani 2003). We retained those gene sets with a *q* value below 0.2, meaning that we expect 20% of our final candidates to be false positives.

#### Jackknife Approach to Detect Pathways with Outlier Genes

We used a jackknife approach to examine the effect of individual genes of a given gene set on our results. For each significant pathway, we repeatedly removed one gene and recalculated the DN/DS and PN/PS values. These jackknife scores are depicted in figures 1*B*–*H* and 2*B*–*E.* Pathways where one of the jackknife scores resulted in a much higher *P* value were probably scoring significant because one gene has extreme values and such pathways were not considered as candidate gene sets for polygenic selection.

#### Classical MDK Test at Gene Set Level

The classical MDK test for positive selection tests at the gene level whether the DN/DS ratio is significantly larger than the PN/PS ratio. We extended this procedure to the gene set level by creating for each gene set a 2 × 2 contingency table containing its DN_set_, DS_set_, PN_set,_ and PS_set_ counts as defined in equation (1) and testing whether the odds ratio of the table (OR = (DS × PN)/(DN × PS) is significantly deviating from (smaller than) one with a two-sided (one-sided) Fisher's exact test, using the R function *fisher.test*.

#### Comparing Gene Set Level Alpha Values with an Empirical Null Distribution

We calculated a gene set analog of α for each set, here defined as

(3) $$\alpha =1-\frac{\sum_{g\in \text{set}}{\text{DS}}_{g}\cdot \sum_{g\in \text{set}}{\text{PN}}_{g}}{\sum_{g\in \text{set}}{\text{PS}}_{g}\cdot \sum_{g\in \text{set}}{\text{DN}}_{g}}$$

and compared it to the α values of an empirical null distribution with corresponding set length.

## Results

### Genes in Biological Pathways Are Conserved and Share Evolutionary Properties

We first investigated whether genes in pathways have different evolutionary properties than genes that are not part of a pathway. We downloaded a collection of 1,366 pathways from the Biosystems database (Geer et al. 2010) and contrasted the genetic diversity of genes that occur in one or more gene sets with genes that are not part of any gene set. We used SNPs inferred from the Complete Genomics (CG) collection of 42 human individuals from three African and five non-African populations (Drmanac et al. 2010), as well as the fixed differences between humans and chimpanzees that were assigned to the human branch. Interestingly, we find that the nonsynonymous to synonymous ratios are lower for genes in gene sets than for genes that are not part of a gene set, both for mutations within human populations (PN/PS) and for fixed mutations on the human lineage (DN/DS). This suggests that genes belonging to gene sets are globally under more severe evolutionary constraints (table 1).

In addition, we find that genes that are part of many pathways (ten or more) are even more constrained than genes occurring in a single or a few pathways, as they show significantly lower DN/DS and PN/PS ratios (*P* < 1e-6, Mann–Whitney test, supplementary fig. S3, Supplementary Material online). Overall, there is a small but significant negative correlation between the DN/DS ratio of a gene and the number of pathways it belongs to (Pearson’s *r* = −0.09, *P* < 2.2e-16). These results make sense, since genes that are part of many pathways often have an essential role or have several functions, suggesting a potential pleiotropic effect of mutations in these genes, and thus a higher chance for them to be deleterious. These findings are also consistent with earlier reports showing that highly connected proteins are usually more essential (Jeong et al. 2001; Wuchty 2004) or evolving at a slower rate (Fraser et al. 2002; Saeed and Deane 2006). These results further imply that tests of selection bearing on single genes should be performed separately for genes not part of gene sets or that their lower levels of evolutionary constraints be taken into account in future testing procedures to minimize false positives.

**Table 1:** Counts and Ratios of Nonsynonymous (*N*) and Synonymous (*S*) Fixed Mutations and Polymorphisms in the Human Lineage

*Mutation Group*	*N*	*S*	*N*/*S*
Divergence (D)			
All genic substitutions	32,332	41,596	0.78
Substitutions in genes belonging to a gene set	13,802	20,811	0.66
Substitutions in genes not belonging to a gene set	18,530	20,785	0.89
Polymorphism (P)			
All genic SNPs	63,812	57,672	1.11
Private African SNPs	27,413	24,953	1.10
Private non-African SNPs	19,965	13,990	1.43
Shared SNPs	16,434	18,729	0.88
SNPs in genes belonging to a gene set	28,876	28,704	1.01
Private African SNPs	12,309	12,377	0.99
Private non-African SNPs	9,263	6,911	1.34
Shared SNPs	7,304	9,416	0.78
SNPs in genes not belonging to a gene set	34,936	28,968	1.21
Private African SNPs	15,104	12,576	1.20
Private non-African SNPs	10,702	7,079	1.51
Shared SNPs	9,130	9,313	0.98

Interestingly, we observe that gene-specific DN/DS and PN/PS ratios are more similar within than between gene sets and that the variance in *N*/*S* ratios due to differences between gene sets represents around 5% (supplementary fig. S2, Supplementary Material online) of the total variance in *N*/*S* ratios (nonparametric ANOVA test, *P* value < 1e-4; see Materials and Methods and supplementary text S1, Supplementary Material online). This variance component also represents the average correlation between *N*/*S* ratios of two genes belonging to the same gene set relative to two genes from different gene sets. This positive correlation of about 5% suggests that genes in a pathway share evolutionary properties and have a correlated evolutionary response (i.e., most are rather conserved or most are under weak selection). These results are in keeping with the observation of Fraser et al. (2002) that interacting proteins have similar evolutionary rates, which the authors explained by the occurrence of compensatory changes in interacting proteins.

### Measuring and Testing Selective Constraints at the Pathway Level

We are interested here in finding a way to determine the extent and the type of selection acting in gene sets. Conventional MDK tests such as those based on the α statistic are usually used to detect only positive selection (but note that a negative α is an indicator of purifying selection or of slightly deleterious mutations segregating in a population). Furthermore, α is based on a ratio of ratios, and a given high α value, taken to be indicative of positive selection, can be obtained with a high DN/DS or a low PN/PS, which can be due to different selective forces, such as recent strong purifying selection. To address these issues, we propose here a new test of polygenic selection that is more informative about the respective importance of DN/DS and PN/PS ratios and that detects outlier pathways for different types of selection. Our aim with this test, referred to hereafter as the 2DNS test, is to find gene sets that have evolved differently than other gene sets, in that they have unusual DN/DS or PN/PS combinations compared with other sets. First, we sum up the PN, PS, DN, and DS counts over all genes in a gene set to obtain gene set level DN/DS and PN/PS ratios (as shown in eq. [1]). Second, we create genome-wide empirical null distributions of random gene sets that take into account gene set size, as well as potentially shared evolutionary properties of gene sets. Note that the use of these null distributions also allows us to control for the past demographic history shared among all gene sets and for the overall selective constraint acting on coding regions. Third, the joint probability density distribution of DN/DS versus PN/PS ratios is estimated on a 2D grid directly from the empirical null distribution. The *P* value for each gene set is finally obtained by integrating the joint density of all grid points that have a similar or lower density than the gene set of interest defined by its “*N*/*S* coordinates” (see supplementary fig. S6, Supplementary Material online).

We compared the distribution of gene set *P* values with a uniform distribution on a QQ plot to confirm that our testing procedure was well behaved (supplementary fig. S5*D*, Supplementary Material online). The fact that the observed *P* values are close to the expected uniform distribution suggests that our null distribution correctly represents the properties of the gene sets. Note that naively constructed null distributions that ignored some or all of the evolutionary properties of gene sets led to strongly underestimated *P* values, which would have translated in a large number of false-positive gene sets (see supplementary S5*A*–*C*, Supplementary Material online).

We applied the 2DNS test to the comparison between fixed mutations on the human branch and polymorphisms that are found both in African and non-African populations. These “shared” polymorphisms come from relatively ancient mutations that predated the migration out of Africa, and we thus expect that slightly deleterious mutations, which can distort selective signals (Eyre-Walker 2002; Fay et al. 2002; Parsch et al. 2009), have been purged from this group. Indeed, the distributions of the GERP score, a measure of evolutionary constraint (Cooper et al. 2005), for fixed mutations and shared polymorphisms are very similar, confirming that the latter are not enriched with deleterious mutations (supplementary fig. S7, Supplementary Material online).

The 2DNS test identifies six gene sets with a *q* value < 0.2 (fig. 1 and table 2). These gene sets are located well outside the bulk of the 2D null distributions in different directions (fig. 1*B*–*G*), suggesting that they are subject to different forms of selection than the majority of sets that are under slightly purifying selection. For each of the significant pathways, we repeatedly recalculated the DN/DS and PN/PS values leaving one gene out in turn (“jackknifing”) to examine to which extent the score depends on single genes (fig. 1*B*–*H*).

**Table 2:** Pathways Scoring a q Value < 0.2 in the 2DNS Test Comparing Fixed Mutations in the Human Branch (DN/DS) and Polymorphisms Shared Between African and non-African Populations (PN/PS)

Rank	Gene Set	Size (Genes)	Length (kb)	DN	DS	DN/DS	PN	PS	PN/PS	*P*	*q*
1	Olfactory transduction	291	425	628	375	1.67	592	323	1.83	9.49e-59	1.06e-55
2	Olfactory Signaling Pathway	250	290	548	298	1.84	532	274	1.94	9.99e-43	5.58e-40
3	Glutathione conjugation	22	78	19	14	1.36	27	9	3.00	5.55e-04	0.1513
4	Beta-catenin phosphorylation cascade	17	109	24	22	1.09	1	12	0.08	6.63e-04	0.1513
5	Meiotic Recombination	57	171	127	88	1.44	26	34	0.76	7.41e-04	0.1513
6	mRNA Splicing*	101	437	41	233	0.18	9	75	0.12	8.13e-04	0.1513

Note.—Gene set length, total exon length of genes in set. Pathways marked with a "*" represent a union of highly similar pathways.

### Significant Pathways Have Been Influenced by Different Types of Selection

The two highest scoring sets, *Olfactory transduction and Olfactory Signaling Pathway*, have both high DN/DS and high PN/PS ratios (fig. 1*B* and *C*), in line with their known relaxed constraints in primates, in particular in humans (Gilad et al. 2005; Somel et al. 2013; Hughes et al. 2014). This relaxation is often explained by the fact that vision has become more important than smell in primates (Gilad et al. 2004). Note that having both DN/DS and PN/PS ratios around two suggest that nonsynonymous mutations are neutral in these genes, since most mutations on the first two codon bases result in nonsynonymous changes. This result is consistent with the observation that many olfactory genes have become pseudogenes (Go and Niimura 2008).

The third high scoring gene set is *Glutathione conjugation*. This pathway contains many glutathione S-transferases (GSTs), which are involved in the protection of cells against oxidative stress and toxic foreign compounds (Hayes and Strange 2000). The jackknife scores of this pathway show that PN/PS ratios are typically high for all genes in this set, with some variation, whereas their DN/DS ratios is very stable and maintained at a moderately high level (fig. 1*D*). The fact that this pathway has an unusually high PN/PS ratio for shared SNPs could be due to some form of balancing selection. It suggests that the high diversity in GSTs is beneficial, possibly serving as a population wide protection against a large range of toxic factors in the environment. Indeed, GST polymorphisms have been related to drug sensitivity and are associated with disease susceptibility (Hayes and Strange 2000).

The next highest scoring pathway is the *Beta-catenin phosphorylation cascade* pathway. Beta-catenin is involved in both cell adhesion and Wnt signaling (signal transduction). It also plays an important role during development and it can act as oncogene (Bienz 2005). This pathway is a good example of having an unusual combination of *N*/*S* coordinates, with PN/PS being much lower than DN/DS, while the separate ratios are not extreme outliers by themselves. This pattern is compatible with a recent strong purifying selection in humans resulting in very low PN/PS values (fig. 1*E*), possibly after an initial adaptive event. Indeed, this is confirmed by the jackknife scores that show a constantly low PN/PS score across all genes. Two genes appear as outliers in opposite directions: The *APC* gene (a gene involved in tumor suppression and in synapse assembly, Rosenberg et al. 2008) plays a large role in the high DN/DS ratio of this gene set, whereas the *AXIN* gene has the opposite effect. When one or the other is removed, the DN/DS ratio changes strongly, but when both are removed, the DN/DS ratio (1.2) is close to the original value (1.25). We therefore posit that the signal shown by this pathway is indeed polygenic and not driven by the effect of a single gene.

The *Meiotic Recombination* pathway is another candidate for being affected by positive selection, with a relatively high DN/DS ratio and a moderate PN/PS ratio (fig. 1*F*). Many genes in this pathway code for histones, but the high DN/DS ratio is caused by other genes, such as *BRCA1*, *MSH4*, *TOP3A*, and *PRDM9* (supplementary table S3, Supplementary Material online), some of which were recently reported to be under positive selection (Lou et al. 2014; Schwartz et al. 2014). Although meiotic recombination is an essential and therefore conserved process in mammals, some of the underlying genetic factors (gene variants, recombination rate, and hotspots) vary among species (Baudat et al. 2013). The fact that *PRDM9*, one of the key players meiotic recombination, has been related with interspecific intercompatibilities (Oliver et al. 2009) suggests that positive selection in the *Meiotic Recombination* pathway could have been involved in the emergence of barriers to reproduction with related species.

The *mRNA Splicing* pathway is a good candidate for being under extremely strong purifying selection as it shows particularly low values both for DN/DS and for PN/PS (fig. 1*G*). This pathway is an extreme example of a “housekeeping” pathway, with genes expressed in all tissues. Earlier studies have indeed reported that housekeeping genes evolve more slowly than tissue specific genes and that they are under stronger selective constraints (e.g., Zhang and Li 2004).

### Impact of Differential Demography in African and Non-African Populations

Previous studies have shown that populations outside Africa have a higher proportion of deleterious mutations than those in Africa (Lohmueller et al. 2008; Subramanian 2012), compatible with the buildup of a mutation load since the expansion of modern humans out-of Africa (Peischl et al. 2013). We examined PN/PS ratios for SNPs that were either shared between Africans and non-Africans or private to Africans or private to non-Africans. In line with previous results (Lohmueller et al. 2008; Peischl et al. 2013), we find that the PN/PS ratio is larger for non-African-specific SNPs than for SNPs private to Africans. In addition, shared SNPs show the lowest PN/PS ratio and this for genes belonging or not to gene sets (table 1). These results are consistent with the view that bottlenecks and range expansions of non-African populations have increased their PN/PS ratio relative to Africans (Peischl et al. 2013) and that purifying selection had more time to act on shared SNPs than on population specific SNPs, thus contributing to the elimination of a proportionally larger number of deleterious nonsynonymous mutations (see also supplementary fig. S7, Supplementary Material online).

To study the effect of potentially different demographic histories in Africans and non-Africans at the gene set level, we used our 2DNS test to compare the PN/PS ratios of private non-African SNPs and private African SNPs. As expected, PN/PS ratios are overall clearly larger for non-African SNPs than for African SNPs at the gene sets level (fig. 2, sign test: *P* < 2.2e-16). In addition to showing the effect of past demography on the whole genome, our procedure reveals four significant outlier pathways (supplementary table S4, Supplementary Material online), which all have unusual *N*/*S* combinations given the genomic background. These pathways are related to olfactory signaling and mRNA processing and they are examples of relaxation and purifying selection, as found in the previous test where we contrasted fixed mutations against shared SNPs.

### Effect of Increasing Sample Size: 2DNS Applied on the 1000 Genomes Dataset

Increasing the number of sampled individuals will not only lead to a higher number of detected SNPs but also to an enrichment in low frequency variants that are potentially under stronger purifying selection. To study this effect, we repeated our testing procedure on another dataset, namely SNPs from the 1000 Genomes project phase 3 (1000G set), using data collected from 1,594 individuals. To properly compare the results, we restricted our analyses to the part of the exome that is covered by both the Complete Genomics and the 1000 Genomes projects. First, we applied the 2DNS test on both SNP sets to compare *N*/*S* ratios between fixed mutations in the human lineage and SNPs shared between African and non-African populations. Although there were twice as many shared SNPs in the 1000G set as in the CG set (supplementary table S5, Supplementary Material online), the top scoring gene sets are very similar (supplementary table S6, Supplementary Material online). Indeed, the four significant gene sets in the CG dataset are the top four of five significant gene sets in the 1000G results. Next, we applied the test on both datasets to compare *N*/*S* ratios between SNPs private to African and non-African populations. Although three out of the four significant sets in the CG results are also significant in the 1000G test, the latter test produces twice as many significant sets in total (supplementary table S7 and fig. S8, Supplementary Material online). This larger number of significant sets seems both due to the much larger number of SNPs in the 1000G set (five times more African SNPs and ten times more non-African SNPs, supplementary table S5, Supplementary Material online) and to the enrichment of slightly deleterious nonsynonymous mutations among rare variants that can inflate the PN/PS ratio. Two high scoring pathways are particularly interesting, because the PN/PS ratio of their African SNPs is larger than the PN/PS ratio of their non-African SNPs. The *Signaling by constitutively active EGFR* pathway has a moderate African PN/PS ratio and lower non-African PN/PS ratio, suggesting recent adaptation in Africa or purifying selection outside Africa. The *Linoleic acid metabolism* pathway has a high PN/PS ratio in both African and non-African SNPs which rather points to a recent relaxation of purifying selection in African populations.

### Comparison between 2DNS and Classical MDK Tests

To illustrate the difference between our new approach and classical MDK tests, we performed an MDK test at the gene set level. In other words, we tested whether some gene sets presented a DN/DS ratio larger than their PN/PS ratio. We inferred the significance of the results in two ways: With a conventional Fisher's exact test on the 2 × 2 contingency table of gene set level DN, DS, PN and PS counts and by comparing the gene set α value computed according to equation (3) (see Materials and Methods) to an empirical null distribution built along the same principles as for the 2DNS test.

With a two-sided Fisher's exact test (testing for deviation from DN/DS = PN/PS) we find 68 pathways scoring significant (*q* value < 20%). Most of them have a PN/PS > DN/DS, which would point toward purifying or balancing selection rather than to positive selection (supplementary table S8, Supplementary Material online). Interestingly, nine out of the ten highest scoring significant sets are directly related to immunity or response to pathogens. There are only four significant pathways with DN/DS > PN/PS, indicative of positive selection, namely *IL-2 Signaling Pathway, IL2-mediated signaling events, Beta-catenin phosphorylation cascade, *and* Pentose phosphate pathway (Pentose phosphate cycle)*, all ranked in the lower half of the list. However, applying a one-sided Fisher's exact test, where we explicitly try to detect cases where DN/DS > PN/PS, produces no significant pathways at FDR level 20% (supplementary table S9, Supplementary Material online). We note, however, that a QQ plot analysis of the *P* values of both Fisher's exact tests shows clear systematic departures from expectations. Indeed, the *P* values are under- and overestimated for the two sided and for the one-sided Fisher exact test, respectively (supplementary fig. S9, Supplementary Material online), implying that the two-sided test is too liberal, and the one-sided test is too conservative.

The comparison of gene set α values to an empirical null distribution results in several high scoring pathways, but none of them are significant after correcting for multiple tests (supplementary table S10, Supplementary Material online), suggesting that a test based on α values is less powerful than our 2DNS test. Moreover, we notice that most of the top-ranking pathways (shown as dark red points on supplementary fig. S10, Supplementary Material online) have a high α value associated with a low DN/DS and an even lower PN/PS. This suggests that positive selection is more easily detected in (or is more likely to act on) slowly evolving genes.

## Discussion

**Fig. 3.—:**
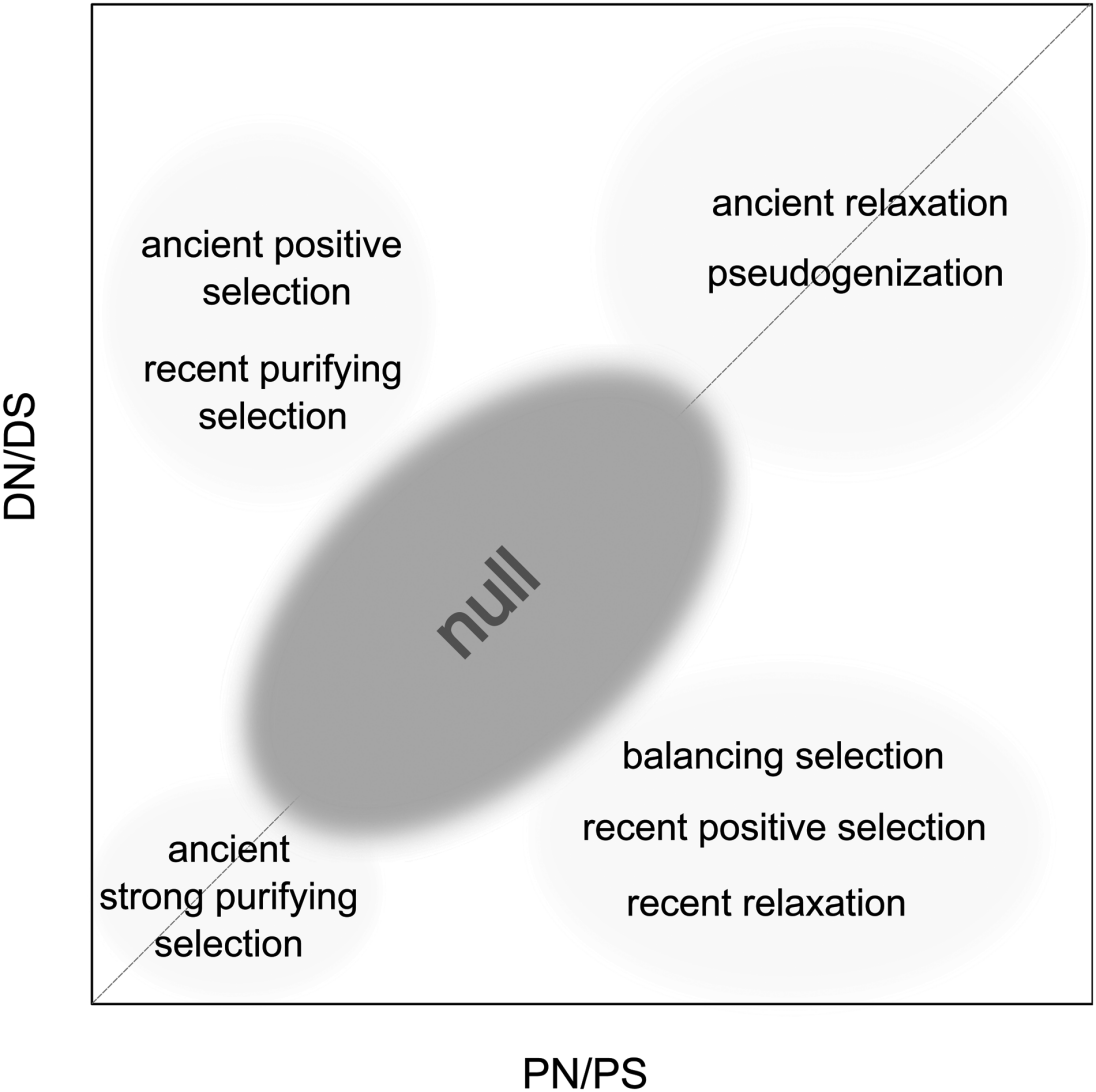
Potential selective forces having acted on significant outlier gene sets depending on their position in the *N*/*S* plane.

Our 2DNS test has several advantages over classical methods such as the MDK test. First, we focus on the detection of selection in functional groups of genes instead of single genes. Not only is a biological pathway or gene network a more natural unit to test for selection, but by pooling genes belonging to the same gene set, we avoid the exclusion of many genes that have undefined *N*/*S* ratios. Second, our test allows one to detect different selective regimes, whereas classical tests are often designed to evidence only one type of selection, usually positive selection. As proposed in figure 3, we can infer which selective regime could have acted on an outlier gene set from its position in the PN/PS-DN/DS plane. Still, different selective processes can yield similar patterns leading to ambivalent interpretations. However, we see this as a problem of the underlying biology (different processes generate similar patterns) rather than of the 2DNS test per se. In these cases, one could inspect the function of the genes of a candidate pathway in more detail to gain insight on the type of selection that might have acted on the gene set.

Our 2DNS test is similar in spirit to the 2D neutrality test proposed by Innan (2006), which incorporates two summary statistics to test genes for signals of selection. However, the statistics used by Innan are indirect measures of neutrality, and the interpretation of these pairs of values is not straightforward. Rather, instead of combining two selection tests, we decompose here the MDK test into its two constituent factors, DN/DS and PN/PS, which allows us to gain more insight into underlying evolutionary pressures.

A third important advantage of our method is that it compares gene sets against a genome-wide empirical null distribution and thus controls for potential demographic events (similar to e.g., Nielsen et al. 2005) and genome-wide selective forces, most notably background selection (Hernandez et al. 2011). It has indeed been noticed that large genomic datasets contain their own null distribution (Efron 2013), implying they can be used to evidence outliers. Note, however, that our approach does not test against any prespecified evolutionary model, and it is therefore not a test of neutrality since gene sets found to be nonsignificant by our approach can be under selection. With our 2DNS test, we are thus trying to find extreme outliers, such as gene sets that have atypical patterns of diversity compared with other gene sets, which makes it a rather conservative method, as we build a null distribution that closely reflects the properties of the gene sets themselves. However, we have shown that using a naïve null distribution, for example, by simply randomly sampling from all genes, would yield many false positives (supplementary fig. S5, Supplementary Material online), because this null distribution would not reflect the inherent properties of gene sets in general. This is especially true because pathways described in current databases are probably a biased subset of existing biological pathways.

In line with earlier studies (Bustamante et al. 2005; Chimpanzee Sequencing and Analysis Consortium 2005; Zhang and Li 2005; Eyre-Walker and Keightley 2009; Messer and Petrov 2013), we find more evidence for purifying rather than positive selection, reflected for example in lower DN/DS ratios than PN/PS ratios, both at the gene and gene set level (table 1). However, a possible explanation for finding only a few examples of positive selection is that it does not affect mostly coding regions, but rather regulatory regions or other functional parts of the genome, leading to variation in gene expression or epigenetic differences (Ponting and Lunter 2006; Enard et al. 2014). The 2DNS test could be adapted to study such categories of genomic data, for example, comparing mutations in transcription factor binding sites to putative neutral flanking sites, as other metrics than synonymous or nonsynonymous state can be used to assign the level of functional or selective constraints; for example, site conservation scores (e.g., GERP scores, Cooper et al. 2005).

Our 2DNS test is not restricted to finding gene sets with unusual DN/DS-PN/PS pairs in the human branch and can easily be extended to other species or applied to compare evolutionary rates between species. The test is also suitable to compare the diversity levels among polymorphisms between groups with a different demographic history, as exemplified in figure 2, where we compare SNPs private to African and to non-African populations.

In the NS plane, many small gene sets show very unusual joint *N*/*S* ratios (fig. 1*A*), but they are not found significant because their associated null distributions are very wide (e.g., fig. 1*H*). This shows that one should be cautious with groups of genes that have high *N*/*S* ratios, as such values can arise by chance alone, but it also suggests that our test might lack power to detect outlier selection regimes in small gene sets. Indeed, when we use a smaller dataset by restricting it to the 86.8% of the exome that is covered by both the polymorphism and divergence datasets (see also Mapping and annotation of mutations in the Materials and Methods section), we get similar top scoring gene sets (supplementary table S11, Supplementary Material online), but they have a lower significance, probably due to the loss of data (20% less SNPs and 10% less fixed mutations).

In addition, it is likely that selection has not affected whole pathways but only some subsets and that this limited signal might be difficult to detect at the whole pathway level. Alternatively, different selective forces might also act on distinct subsets of genes, and their signal could cancel each other’s out when examining whole pathways, leading to a reduced power of our approach. Note that this problem also occurs when testing single genes, since different exons, introns, or regulatory regions can be affected by different selective forces. It would thus be useful to extend our test to detect selection in pathway submodules, which should be the object of future work.

Our previous attempt at detecting selection at the gene set level within human populations revealed several pathways involved in immune response to be potentially under positive selection (Daub et al. 2013). Interestingly, our present study does not show strong signals of positive selection but rather of overall purifying selection. These differences can be due to several reasons. First, our previous study used a statistic (hierarchical *F*_ST_) detecting differences between continental groups that should have emerged recently, whereas our 2DNS statistics are more sensitive to older events. We contrast old mutations that have occurred on the human lineage to polymorphisms shared between Africans and non-Africans (fig. 1) and which should thus have occurred in the ancestral human populations prior to the exit out of Africa. Second, our current testing procedure is more stringent than that used previously. We are indeed looking for outliers in a null distribution that takes into account selective constraints acting on pathways and past demographic effects, whereas we previously based our test on a null model of human evolution only taking into account global levels of differentiation between human populations. Third, we focused here strictly on coding regions, whereas we also considered neighboring regions in our previous study, thus including regulatory and enhancer regions that have been recently shown to bear the strongest signals of positive selection in humans (Enard et al. 2014). These differences in methodology and in the investigated time scales could thus explain the apparent discrepancies found between these two studies. This also indicates that different forces have acted on pathways at different periods of human evolution.

## Supplementary Material

Supplementary text S1 and S2, tables S1–S11, and figures S1–S10 are available at *Genome Biology and Evolution* online (http://www.gbe.oxfordjournals.org/).
